# Clinical profile and molecular characterization of Galactosemia in Brazil: identification of seven novel mutations

**DOI:** 10.1186/s12881-016-0300-8

**Published:** 2016-05-12

**Authors:** Daniel F. Garcia, José S. Camelo, Greice A. Molfetta, Marlene Turcato, Carolina F. M. Souza, Gilda Porta, Carlos E. Steiner, Wilson A. Silva

**Affiliations:** Department of Genetics, Ribeirão Preto Medical School, University of São Paulo, Ribeirão Preto, SP Brazil; Department of Pediatrics, Ribeirão Preto Medical School, University of São Paulo, Ribeirão Preto, SP Brazil; Department of Neurology, Ribeirão Preto Medical School, University of São Paulo, Ribeirão Preto, SP Brazil; National Institute of Science and Technology in Stem Cell, and Cell Therapy, Regional Blood Center of Ribeirão Preto, Ribeirão Preto, SP Brazil; Department of Genetics, Clinical Hospital of the Porto Alegre, Porto Alegre, RS Brazil; Department of Pediatrics, Children’s Institute, Medical School of the University of São Paulo, São Paulo, SP Brazil; Department of Medical Genetics, School of Medical Science, State University of Campinas, Campinas, SP Brazil; Center for Medical Genomics at Clinical Hospital of the Medical School of Ribeirão Preto, University of São Paulo, Ribeirão Preto, SP Brazil

**Keywords:** GALT, Inborn error of galactose metabolism, Mutation screening

## Abstract

**Background:**

Classical Galactosemia (CG) is an inborn error of galactose metabolism caused by the deficiency of the galactose-1-phosphate uridyltransferase enzyme. It is transmitted as an autosomal recessive disease and is typically characterized by neonatal galactose intolerance, with complications ranging from neonatal jaundice and liver failure to late complications, such as motor and reproductive dysfunctions. Galactosemia is also heterogeneous from a molecular standpoint, with hundreds of different mutations described in the *GALT* gene, some of them specific to certain populations, reflecting consequence of founder effect.

**Methods:**

This study reviews the main clinical findings and depicts the spectrum of mutations identified in 19 patients with CG, six with Duarte Galactosemia and one with type 2 Galactosemia in Brazil. Some individuals were diagnosed through expanded newborn screening test, which is not available routinely to all newborns.

**Results:**

The main classical Galactosemia mutations reported to date were identified in this study, as well as the Duarte variant and seven novel mutations - c.2 T > C (p.M1T), c.97C > A (p.R33S), c.217C > T (p.P73S), c.328 + 1G > A (IVS3 + 1G > A), c.377 + 4A > C (IVS4 + 4A > C), c.287_289delACA (p.N97del) and c.506A > C (p.Q169P). This was expected, given the high miscegenation of the Brazilian population.

**Conclusions:**

This study expands the mutation spectrum in *GALT* gene and reinforces the importance of early diagnosis and introduction of dietary treatment, what is possible with the introduction of Galactosemia in neonatal screening programs.

**Electronic supplementary material:**

The online version of this article (doi:10.1186/s12881-016-0300-8) contains supplementary material, which is available to authorized users.

## Background

The ubiquitously expressed enzyme galactose-1-phosphate uridyltransferase (GALT; EC 2.7.7.12) is a component of the galactose metabolism pathway that catalyzes the conversion of galactose-1-phosphate with UDP glucose to glucose- 1-phosphate and UDP galactose [1, 2]. Its deficiency causes classical galactosemia [CG; MIM#230400] [3], which is presented in the neonatal period as poor feeding, vomiting, diarrhea, jaundice, liver and renal failure, hypoglycemia, muscular hypotonia, sepsis and cataract [4, 5]. The treatment is the diet restriction of lactose-containing foods, which reverses the majority of neonatal symptoms, but does not prevent late complications such as impairment of mental development, disorders of speech and motor function, and reproductive system abnormalities in some cases [6–14]. The cDNA of the gene that encodes GALT (NG_009029.1) was cloned and characterized by Reichardt and Berg (1988) [15], it is located on chromosome 9p13 [16] with approximately 4 kb of DNA sequence arranged into 11 exons. The CG is inherited as an autosomal recessive disease and 336 different mutations have been identified so far [17].

Classical Galactosemia is heterogeneous at clinical and molecular level, but there are some common mutations, as the c.563A > G (p.Q188R) and the c.855G > T (p.K285N) [18–20] in Caucasian populations and the c.404C > T p.S135L [21, 22] in individuals of African origin. Besides the deleterious mutations, the most common *GALT* mutant is Duarte 2 (D2) allele, characterized by some sequence changes: a c.940A > G (p.N314D) missense substitution, three intronic base changes and a 4 bp deletion in the 5′ proximal sequence [23–25]. The D2 allele in heterozygous with one allele of CG causes a mild type of galactosemia, called Duarte galactosemia. Apart from GALT deficiency galactosemia, there are also other rare types of galactose metabolism diseases, Type II Galactosemia (OMIM 230200), caused by deficiency of the enzyme Galactokinase (GALK, EC 2.7.1.6), characterized by early onset bilateral cataract and some neurological manifestations [26], and type III Galactosemia (OMIM #230350) caused by mutations in *GALE* gene (EC 5.1.3.2) leading to UDP-galactose 4-epimerase deficiency [27].

The aim of this study is to describe the profile of mutations in the *GALT* gene of the Brazilian patients with CG and for newborns that present positive galactosemia newborn screening test, in addition to studying the genotype-phenotype correlation. This study provides some information for discussions about the introduction of Galactosemia in the national newborn screening program in Brazil, where the prevalence of CG is estimated close to 1:20,000 [28].

## Methods

### Patients and ethical aspects

Thirty patients (60 alleles), including two sib pairs, who have the diagnosis of Galactosemia confirmed by biochemical analysis, were analyzed. The patients come from the Clinical Hospital of the Ribeirão Preto Medical School, University of São Paulo (8 patients) and other services and hospitals in Brazil (22 patients). Six patients of the study were diagnosed through expanded newborn screening test, which is not available as routine for all newborns in Brazil. Clinical data of patients were obtained from a review of the medical records, using a standardized form. The Research Ethics Committee of the Hospital approved the study and a written informed consent was obtained from each patient or responsible family member.

### Red blood cell GALT assay

The GALT enzyme activity was detected by an enzymatic-fluorometric method [29]. The fluorescence reading at 460 nm was obtained with a Hitachi F-2000 fluorometer (Hitachi, Tokyo, Japan) and to measure haemoglobin concentration, an absorbance reading at 410 nm was obtained with a Hitachi U-2001 spectrophotometer (Hitachi, Tokyo, Japan). The normal range was defined as 37– 66 μmol/h per gHb.

### DNA amplification and exon sequencing

All patients were subjected to exons sequencing of the *GALT* gene. In order to diagnose a possible galactosemia due to galactokinase deficiency, one patient with elevated total galactose and normal erythrocyte *GALT* enzyme activity also underwent *GALK1* gene exons sequencing.

Genomic DNA was extracted from peripheral blood leukocytes, using a Super Quick-gene-rapid DNA isolation kit (Promega, Madison, WI, USA), following the manufacturer’s instructions. Eight pairs of primers were designed to amplify the promoter region, the 11 exons and adjacent intronic regions of the *GALT* gene. For the analysis of the *GALK1* gene, six pairs of primers were designed to cover the eight exons and their respective splice site junctions (primer sequences and PCR conditions in Additional file 1).

The PCR-amplified DNA fragments were subjected to direct sequencing in an automatic capillary sequencing system ABI 3130 Genetic Analyzer (Applied Biosystems, Foster City, CA, USA), using the Big Dye® terminator v3.1 cycle sequencing kit (Applied Biosystems, Foster City, CA, USA) and the same PCR primers, following the manufacturer’s instructions. The results were analyzed using the FinchTV version 1.4.0 (Geospiza, Seattle, WA, USA) and Codoncode Aligner (Codoncode, Centerville, MA, USA). The sequences obtained were compared with the reference ones from GenBank database (NG_009029.1/NM_000155.3 and NG_008079.1/NM_000154.1).

### In silico analysis

In order to predict damage effects of missense and splice site mutations, we performed *in silico* simulations using the following bioinformatics tools: 1) SIFT [30], that classify variants according to mathematical operations; 2) PolyPhen2 [31], that uses Bayesian methods, and; 3) BDGP Splice Site Prediction software, a system that evaluate changes in splice site strength based on by Neural Network models [32].

## Results

The average age of diagnosis was about one and a half month, excluding from this calculation the patient with 48 years of old. During the review of patients’ records and analyzing the standardized form data of patients from other participating centers, it was observed as the main clinical findings: hepatomegaly, jaundice, hemolytic anemia, failure to thrive and bilateral cataract. Some patients show developmental delay and the only adult patient included has primary ovarian insufficiency (Table 1).

**Table 1:** Clinical summary of patients with galactosemia and results of GALT enzyme activity assay, and genotyping

	Age at diagnosis	Age at last evaluation	Hepatomegaly	Jaundice	Vomiting	Failure to thrieve	Cataracts	Hemolytic anemia	Ataxia/Ovarian failure	GALT activity (μmol/h/g Hb)	*GALT* Genotype	Diagnosis
1	2 m	4y	+	+	+	+	-	+	-	Undetectable	p.Q188R/p.Q188R	Classical galactosemia
2	2 m	10 m	+	+	+	+	-	-	-	5.0	p.Q188R/p.Q188R	Classical galactosemia
3	1 m	12 m	+	+	-	-	-	-	-	3.1	p.Q188R/p.Q188R	Classical galactosemia
4	4 m	5 m	+	+	-	-	-	-	-	1.5	p.Q188R/p.Q188R	Classical galactosemia
5	1 m	2 m	+	+	+	-	+	-	-	14.5	p.Q188R/p.K285N	Classical galactosemia
6	1½ m	5y	+	+	-	+	-	-	-	11.0	**IVS3nt + 1G > A**/p.Q188R	Classical galactosemia
7	1 m	16 m	+	+	+	+	-	-	-	Undetectable	**p.Q169P**/p.Q188R	Classical galactosemia
8	NS	2y	-	+	-	+	-	-	-	13.3	**IVS4nt + 4A > C**/p.Q188R	Classical galactosemia
9	½ m	12 m	+	+	+	+	-	-	-	Undetectable	**p.M1T**/p.Q188R	Classical galactosemia
10	2 m	4y	+	-	+	+	-	-	-	Undetectable	p.S135L/p.Q188R	Classical galactosemia
11	1 m	3y	+	+	+	-	+	-	-	Undetectable	p.S135L/p.Q188R	Classical galactosemia
12	NS	5 m	-	+	-	+	-	+	-	Undetectable	p.S135L/p.G175D	Classical galactosemia
13	4½ m	4y	+	+	-	-	+	+	-	Undetectable	p.S135L/p.K285N	Classical galactosemia
14	2 m	2 m	+	+	-	+	-	-	-	Undetectable	p.S135L/p.L275fs	Classical galactosemia
15	1 m	3 m	+	-	+	+	-	-	-	Undetectable	p.S135L/p.F171S	Classical galactosemia
16*	2 m	3 m¢	+	+	-	+	+	-	-	1.5	**p.M1T**/p.S135L	Classical galactosemia
17*	1 m	5y	+	-	-	+	-	+	-	1.0	**p.M1T**/p.S135L	Classical galactosemia
18	3 m	2y	+	+	+	+	-	-	-	12.0	p.R33H/p.S135L	Classical galactosemia
19	1½ m	3y	+	+	+	+	+	+	-	Undetectable	**IVSnt4 + 4A > C**/p.R231H	Classical galactosemia
20	NS	10 m	-	-	-	-	-	-	-	3.0	**p.P73S**/p.N314D§	Duarte galactosemia
21	2 m	3 m	-	+	-	-	-	-	-	10.0	p.G175D/N314D§	Duarte galactosemia
22#	NS	5 m	-	-	-	-	-	-	-	13.0	p.R204X/N314D§	Duarte galactosemia
23#	NS	17 m	-	-	-	-	-	-	-	19.0	p.R204X/N314D§	Duarte galactosemia
24	NS	4 m	-	-	-	-	-	-	-	29.0	p.Q188R/N314D§	Duarte galactosemia
25	NS	2 m	-	-	-	-	-	-	-	29.8	**p.N97del**/p.N314D§	Duarte galactosemia
26	3 m	4y	+	-	-	-	-	-	-	7.0	p.H132Y/p.T292Tp.H315H	Galactosemia allele carrier
27	NS	3 m	-	-	-	-	-	-	-	13.0	p.I170I/p.Q188R	Galactosemia allele carrier
28	48y	48y	-	-	-	-	-	-	+	21.0	**p.R33S**/p.S293S	Galactosemia allele carrier
29	2 m	28 m	-	-	-	-	-	-	-	33.0	WT/p.P325L	Galactosemia allele carrier
30@	NS	3 m	-	-	-	-	-	-	-	47.0	WT/WT	Galactosemia type 2

NS = Newborn screening; m = months; y = years; *sip pair, #sib pair, ¢died at 3 month of age

§ = Duarte allele: c.-119_-116delCAGT + c.508-24G > A + c507 + 62G > A + N314D

*@GALK1* genotype: c.166-5_c.227dup67 and c.766C > T (p.R256W)

New mutations are indicated in bold

**Fig. 1:**
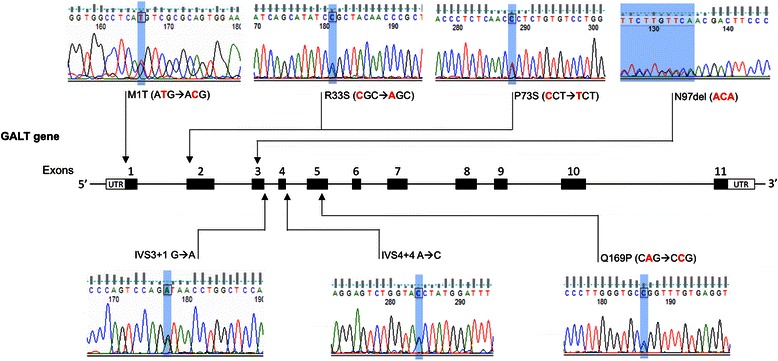
Electropherograms of the novel GALT mutations identified in this study

DNA sequencing analysis identified 18 different pathogenic mutations on the *GALT* gene (Table 2), the two with higher relative allelic frequency were the c.563A > G[p.Q188R] (22 %) and c.404C > T[p.S135L] (12 %). We also identified the Duarte allele and seven new mutations: c.2 T > C[p.M1T], c.97C > A[p.R33S], c.217C > T[p.P73S], c.287_289delACA[p.N97del], c.328 + 1G > A[IVS3 + 1G > A], c.377 + 4A > C[IVS4 + 4A > C] and c.506A > C[p.Q169P] (Fig. 1). Six of these new mutations were in heterozygous with frequent ones (IVS3 + 1G > A/p.Q188R, IVS4 + 4A > C/p.Q188R, p.M1T/p.S135L, p.M1T/p.Q188R, p.P73S/p.N314D, p.N97del/p.N314D and p.Q169P/p.Q188R). Patients who carried new mutations showed reduced or undetectable GALT enzyme activity (Table 1).

**Table 2:** GALT mutations in the Brazilian patients with galactosemia

Region	nucleotide change	Mutation type	Amino Acid	Damage Prediction by SIFT	Damage Prediction by Polyphen-2	Classification@	Relative allele frequency	References
5′UTR	-119_-116delCAGT*	deletion	---	---	---	Benign (Duarte 2)	0,08	Berry GT et al., 2001 [44]
**Exon 1**	**c.2 T > C**	**missense**	**M1T**	**DAMAGING**	**TOLERATED**	**Pathogenic**	**0,04**	**novel**
**Exon 2**	**c.97C > A**	**missense**	**R33S**	**DAMAGING**	**DAMAGING**	**Pathogenic**	**0,01**	**novel**
Exon 2	c.98G > A	missense	R33H	DAMAGING	DAMAGING	Pathogenic	0,01	Gort L et al., 2006 [45]
**Exon 2**	**c.217C > T**	**missense**	**P73S**	**TOLERATED**	**DAMAGING**	**Predicted pathogenicity**	**0,01**	**novel**
**Exon 3**	**c.287_289delACA**	**deletion**	**N97del**	**---**	**---**	**Pathogenic**	**0,01**	**novel**
**Intron 3**	**c.328 + 1G > A**	**splicing efect#**	**---**	**---**	**---**	**Predicted pathogenicity**	**0,01**	**novel**
**Intron 4**	**c.377 + 4A > C**	**splicing efect#**	**---**	**---**	**---**	**Predicted pathogenicity**	**0,03**	**novel**
Exon 5	c.394C > T	missense	H132Y	DAMAGING	DAMAGING	Pathogenic	0,01	Elsas LJ et al., 1998 [46]
Exon 5	c.404C > T	missense	S135L	DAMAGING	DAMAGING	Pathogenic	0,12	Reichardt JK et al., 1992 [47]
**Exon 5**	**c.506A > C**	**missense**	**Q169P**	**DAMAGING**	**DAMAGING**	**Pathogenic**	**0,01**	**novel**
Intron 5	c.508-24G > A*	polymorphism	---	---	---	Benign	0,08	Kozak L et al., 2000 [48]
Intron 5	c.507 + 62G > A*	polymorphism	---	---	---	Benign	0,08	Kozak L et al., 2000 [48]
Exon 6	c.510C > A	silent	I170I	TOLERATED	TOLERATED	Translationally silent	0,01	Item C et al., 2002 [49]
Exon 6	c.512 T > C	missense	F171S	DAMAGING	DAMAGING	Pathogenic	0,01	Reichardt JK et al., 1992 [47]
Exon 6	c.524G > A	missense	G175D	DAMAGING	DAMAGING	Pathogenic	0,03	Gort L et al, 2006 [45]
Exon 6	c.563A > G	missense	Q188R	DAMAGING	DAMAGING	Pathogenic	0,22	Reichardt JK et al., 1992 [47]
Exon 7	c.610C > T	nonsense	R204X	---	---	Pathogenic	0,03	Tyfield L et al., 1999 [35]
Exon 8	c.692G > A	missense	R231H	DAMAGING	DAMAGING	Pathogenic	0,01	Ashino J et al., 1995 [41]
Exon 9	c.824delT	deletion	L275Qfs*5	---	---	Pathogenic	0,01	Elsas LJ et al., 1998 [46]
Exon 9	c.855G > T	missense	K285N	DAMAGING	DAMAGING	Pathogenic	0,03	Leslie ND et al., 1992 [33]
Exon 9	c.876G > A	silent	T292T	TOLERATED	TOLERATED	Translationally silent	0,03	Calderon FR et al., 2007 [17]
Exon 9	c.879C > T	silent	S293S	TOLERATED	TOLERATED	Translationally silent	0,01	Calderon FR et al., 2007 [17]
Exon 10	c.940A > G*	missense	N314D	TOLERATED	TOLERATED	Benign (Duarte 1 and 2)	0,08	Reichardt JK et al., 1991 [50]
Exon 10	c.945 T > C	silent	H315H	TOLERATED	TOLERATED	Translationally silent	0,03	Lai K et al., 1996 [21]
Exon 10	c.974C > T	missense	P325L	DAMAGING	DAMAGING	Pathogenic	0,01	Greber-Platzer S et al., 199 [51]7

*these mutations are found in cis in Duarte 2 allele

#

*@*

New mutations are indicated in bold

Four patients were genotyped as homozygous for the allele p.Q188R, one patient was heterozygous for wild type and p.P325L allele and three were heterozygous for a CG mutation with predicted translationally silent mutations (p.H132Y/p.T292T + p.H315H, p.R33S/p.S293S and p.I170I/p.Q188R). The majority of the patients (22) were genotyping as compound heterozygotes for two of the following mutations: p.M1T, p.R33H, p.P73S, p.N97del, IVS3 + 1G > A, IVS4 + 4A > C, p.S135L, p.Q169P, p.F171S, p.G175D, p.Q188R, p.R204X, p.R231H, p.L275Qfs*5, p.K285N, p.N314D and p.P325L (Table 1).

**Fig. 2:**
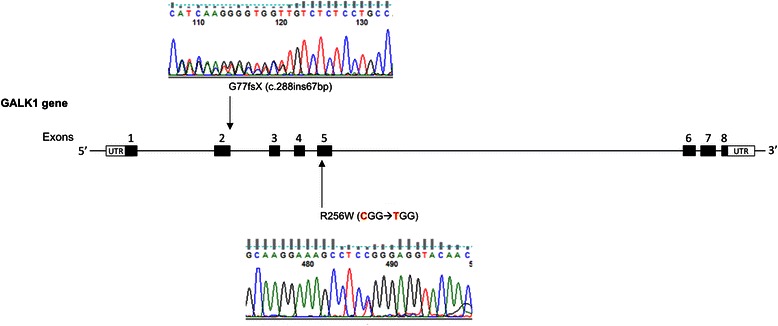
Electropherograms of the GALK1 mutations identified in the patient with galactosemia type II

Interestingly, a patient who has been identified by neonatal screening with high total galactose level had normal GALT activity assay and no mutation in the coding region of *GALT* gene. Further analysis revealed that the patient had type II galactosemia, caused by two mutations in *GALK1* gene: c.166-5_c.227dup67 and p.R256W (Fig. 2).

## Discussion

In order to characterize the molecular basis of the galactosemia in Brazil, we analyzed samples from 30 patients suspected to have galactosemia. The results revealed 26 different *GALT* mutations and two on the *GALK1* gene, with an average rate of pathogenic alleles detection of 93 % (56/60). The p.Q188R mutation was the most frequent (22 %), followed by the p.S135L (12 %) and the Duarte allele (8 %). The mutation p.M1T was found in two independent alleles (three patients, including a pair of brothers), and IVS4 + 4A > C, p.G175D and p.R204X were identified in two alleles each, with p.R204X also present in brothers. The mutations p.M1T, p.R33S, p.P73S, p.N97del, IVS3 + 1G > A, IVS4 + 4A > C and p.Q169P identified in this study have never been previously described. Regarding the new mutations, we looked for these mutations in different databases from normal individuals (1000 genomes, HapMap, dbSNP) and cancer patients (COSMIC). As we did not identify any of them in these databases, we are confident to firm that all the variants are considered novel.

Regarding the phenotype, it was observed a broad clinical spectrum, ranging from a rapidly progressive phenotype with hepatic failure that results in death in the third month of life, to more slowly progressive forms, in which were observed only mild hepatomegaly.

For patients who received the diagnosis after a specific clinical situation, it was possible to establish a genotype-phenotype correlation. Patients who were homozygous for the p.Q188R mutation presented more severe phenotype, as well as the patient with undetectable GALT activity who is compound heterozygous for the mutation p.Q188R and new one p.Q169P. Both mutations are in a highly conserved domain [33, 34]. The p.Q188R mutation is more common in European populations or those predominantly of European origin [35]. Individuals homozygous for p.Q188R allele tend to have a severe phenotype and about 10 % of normal enzymatic activity, as observed in in vitro expression systems [36, 37].

The compound heterozygous patient for mutations p.Q188R and IVS3 + 1G > A also showed a severe phenotype, progressing to cirrhosis before starting treatment. She had a history of a sister who died of liver failure at two months of age of unknown cause. We postulate that the single base change G-to-A that corresponds to a change in the 5′-GU splice donor site of Intron 3 makes the site unrecognizable to the splicing enzymes, with a consequent excision of the exon 3 in the mRNA and production of a truncated protein. It is exactly what happens, for example, with the common mutation of Phenylketonuria IVS12 + 1G > A, that causes skipping of the 12th exon when RNA is being spliced [38]. Some mutations were described at the splice donor site of other introns in CG patients [17, 18, 39].

The proband who had the mutations p.M1T and p.S135L, at the age of two months, showed anemia, hepatomegaly, bilateral cataracts and ascites, progressing in a month to hepatic failure, dehydration and hepatic encephalopathy. She was diagnosed with CG, but her clinical status was so severe that she died due to septic shock, shortly after starting the diet. Her brother, who was born one year after, was diagnosed within the first month of life and began the treatment early. He is currently 5 years old and shows a good clinical outcome, presenting only with a slight enlarged liver and mild bilateral nuclear cataract. The p.M1T mutation was also found in heterozygous with p.Q188R in a patient diagnosed soon after birth with a classic severe phenotype.

The p.M1T should be considered a severe mutation because affects the start codon and might cause out-of-frame usage of the next AUG triplet, 112 nucleotides downstream; it is therefore expected to produce an unstable nonfunctional protein. So the fact that it was considered benign by polyphen-2 although pathogenic by SIFT may be because the polyphen-2 consider structural change that the mutation causes to the protein and does not take into account the fact that the protein is truncated.

Other individuals with a p.S135L mutation had an acute moderate phenotype, which was consistent with what is described in the literature. The p.L135 allele is found almost exclusively in people of African descent and individuals carrying this allele have residual enzyme activity of GALT in some tissues and a less severe phenotype [21, 40].

The only adult patient included in this study was a 48 years woman, presenting with a primary ovarian failure, ataxia and a moderate residual GALT enzyme activity. She had no other symptoms and during the genotyping only the missense new mutation p.R33S and the mutation/polymorphism p.S293S were detected.

Two unrelated patients from different geographical regions of Brazil have the IVS4 + 4A > C mutation. The *in silico* splicing analysis revealed that this mutation abolishes the donor splicing site (donor score <0.40). Therefore, the IVS4 + 4A > C may be considered as a pathogenic mutation. One is a compound heterozygote with the p.Q188R mutation, and the other with the p.R231H mutation. Both showed a good clinical outcome. The first was diagnosed through expanded neonatal screening test and therefore started treatment early. The second one was with jaundice and hepatomegaly at 15 days of age and was diagnosed a month later, when in addition, he presented cataracts. The p.R231H mutation was first described in a Japanese patient, it is predicted to reduce to 15 % of normal controls the GALT enzyme activity in a COS cell expression system [40] and has no detectable activity when expressed in *E. coli* BL21 cells [37], as observed in this patient.

The most common variant of the gene is the Duarte (D2) allele, that is generally not associated with a clinical phenotype when in homozygous, and when in heterozygous with a disease-causing mutation can trigger a mild form of galactosemia called Duarte galactosemia [23]. In this study, six Duarte alleles have been identified (10 %). All individuals with Duarte galactosemia, except one, were diagnosed through expanded newborn screening test, including the compound heterozygotes D2/p.P73S and D2/p.N97del.

The p.P73S mutation is considered tolerable in SIFT and probably damaging by the Poliphen-2. This individual is asymptomatic, even without diet restriction and despite the low enzymatic activity, therefore it is not possible to establish whether this new mutation is pathogenic or not, additional functional studies are necessary.

The only patient with Duarte galactosemia who was diagnosed after a clinical suspicion, is compound heterozygote D2/p.G175D and had only prolonged neonatal jaundice. This patient has an enzyme assay below the normal range and was in a galactose restriction diet.

The patient heterozygous for the p.P325L was asymptomatic but had a positive newborn screening test for galactosemia, the first GALT activity was 12 umol/h/gHb and the second, at two month of age, was 33 umol/h/gHb. As she had a normal total galactose assay (4,3 mg/dl), the breast-feeding was kept and she remained without symptoms.

Definitely, with the availability of neonatal screening test for Galactosemia routinely in some countries, the clinical outcome improved a lot. The greatest benefit derived from this approach is that it is possible to introduce therapy before the onset of acute severe complications. However, it is noteworthy that not every positive test is followed by the diagnosis of CG. Many positive tests are due to variant Duarte and some to other forms of Galactosemia. Therefore, after a positive test, it is important apart from starting treatment, make quickly a careful clinical assessment and perform genotyping, in order to determine whether the patient will be kept in galactose restriction diet.

## Conclusions

Genetic heterogeneity documented to date, undoubtedly contributes to the phenotypic heterogeneity that is observed in galactosemia. Additional effects of non-allelic variation and other constitutional factors on phenotypic variability remain to be elucidated. The present study characterized the phenotypic and genotypic profile of some patients with classic galactosemia in the Brazilian population, which is considered one of the most heterogeneous in the world, as a result of more than five centuries of miscegenation among mainly three ancestral roots: the indigenous Amerindians, Europeans and sub-Saharan Africans. The heterogeneity and admixture have important implications in the current genetic background of the population [42, 43] that has a high estimated prevalence of CG compared with other populations [28]. This is the first genotyping study in Brazilian patients with diagnosis of Galactosemia.

## Ethical approval

All procedures performed in studies involving human participants were in accordance with the ethical standards of the institutional and/or national research committee and with the 1964 Helsinki declaration and its later amendments or comparable ethical standards.

## Additional file

Additional file 1: Table S1. Primer information to sequencing all coding regions of the genes GALT andGALK1. **Table S2**. results of the pathogenic mutation prediction using the SIFT and Polyphen platforms. (DOCX 18 kb)
