# Supplementary material for: Clinical profile and molecular characterization of Galactosemia in Brazil: identification of seven novel mutations
Source: BMC Med Genet. 2016 May 12;17:39. doi: 10.1186/s12881-016-0300-8 (PMC4866286; doi:10.1186/s12881-016-0300-8)
Supplement: Additional file 1: Table S1. — Primer information to sequencing all coding regions of the genes GALT andGALK1. Table S2. results of the pathogenic mutation prediction using the SIFT and Polyphen platforms. (DOCX 18 kb) [file 12881_2016_300_MOESM1_ESM.docx]

**Supplemental material S1**

All PCR reactions were standardized with the following conditions: 2.5 mM of each primer, 2.5 mM of dNTPs, 2.5 µL of 1x buffer (Biotools), 1U of Taq polymerase (Biotools); 200 ng of genomic DNA, and 14 µL of distilled water, in a final reaction volume of 25 µL. PCR was performed as follows: an initial denaturation step of 4 min, followed by 35 cycles consisting of 40 s at 94 °C (denaturation), 50 s at annealing temperatures (specific for each reaction) (Table 2), an extension of 50 s at 72 °C, and a final extension of 10 min at 72 °C.

Table S1-a: Primer sequences and product sizes for PCRs of *GALT* gene.

| Primer | Primer sequence | Amplicon lenght |
| --- | --- | --- |
| GALT_EX1_F | TGAAGTAGGATCATCAATGTCGG | 413bp |
| GALT_EX1_R | GCAGACTGTACGCCTTGTCG |  |
| GALT_EX2_F | AGACCGACAAGGCGTACAGT | 471bp |
| GALT_EX2_R | TGACCCAGAAGGAGGTTCAC |  |
| GALT_EX3/4_F | GCCTTCCCTACTCCCTTGTAG | 371bp |
| GALT_EX3/4_R | CCCAATGCTGAGTCTCCAAC |  |
| GALT_EX5/6_F | GTTGGAGACTCAGCATTGGG | 536bp |
| GALT_EX5/6_R | CCACATCATTGGCATATTTCC |  |
| GALT_EX7_F | GTTTCTTGGCTGAGTCTGAGC | 440bp |
| GALT_EX7_R | CCACTAACCACCCGAGTCTAAG |  |
| GALT_EX8/9_F | GGTGAGAAGACATCAGATCCTG | 531bp |
| GALT_EX8/9_R | GGCTTGCTCCTTTGAGGTT |  |
| GALT_EX10_F | GTGCTTTCTAATCTCCTGCCAG | 432bp |
| GALT_EX10_R | CAGTCCCTTCCTGCCAGAG |  |
| GALT_EX11_F | CCTCCTTAATTGCTCCCTGTC | 461bp |
| GALT_EX11_R | CCTAAACTGGGAATGCTGTCA |  |

Table S1-b: Primer sequences and product sizes for PCRs of *GALK1* gene.

| Primer | Primer sequence | Amplicon lenght |
| --- | --- | --- |
| GALK1_EX1_F | TCATTGGTTCTTCCCGAAGT | 615bp |
| GALK1_EX1_R | CTTCCAACGTGGGGAACAG |  |
| GALK1_EX2_F | GCAGTGTCATTGGAGAGGCT | 355bp |
| GALK1_EX2_R | CCTTCCCCACAGTGTATCAGA |  |
| GALK1_EX3_F | GGTTGGTGGCTTCTGACAAT | 450bp |
| GALK1_EX3_R | CAGCTATTGTGCCCGAGTCT |  |
| GALK1_EX4/5_F | CTGGAGTGTCATTGAAGCCA | 642bp |
| GALK1_EX4/5_R | TTGAAGGGACTGGGGAGAG |  |
| GALK1_EX6/7_F | GGCTGCTGACTCCTCTTTCC | 276bp |
| GALK1_EX6/7_R | GCAGCTCACCTCATAGTCGTCT |  |
| GALK1_EX8_F | CTGTGCCTGGGGTTTATGG | 412bp |
| GALK1_EX8_R | CCAAGCATACACCCACCTCT |  |

**Supplemental material S2**

Results of *In silico* analisys

Table S2-a: Results of SIFT simulations

| **User input Coordinates** | **Codons** | **Transcript ID** | **Substitution** | **Region** | **dbSNP ID** | **SNP/Mutation**  **Type** | **Prediction** | **SIFT Score** | **Median Information Content** |
| --- | --- | --- | --- | --- | --- | --- | --- | --- | --- |
| 9,34646703,1,T/C | ATG-AcG | ENST00000378842 | M1T | EXON CDS | novel | Nonsynonymous | DAMAGING *Warning! Low confidence. | 0 | 3.45 |
| 9,34647100,1,C/A | CGC-aGC | ENST00000378842 | R33S | EXON CDS | novel | Nonsynonymous | DAMAGING | 0 | 2.98 |
| 9,34647101,1,G/A | CGC-CaC | ENST00000378842 | R33H | EXON CDS | [rs111033829:A](http://www.ncbi.nlm.nih.gov/sites/entrez?db=snp&cmd=search&term=+rs111033829) | Nonsynonymous | DAMAGING | 0 | 2.98 |
| 9,34647220,1,C/T | CCT-tCT | ENST00000378842 | P73S | EXON CDS | novel | Nonsynonymous | TOLERATED | 0.12 | 3.01 |
| 9,34647241,1,C/T | CGA-tGA | ENST00000378842 | R80* | EXON CDS | [rs111033664:T](http://www.ncbi.nlm.nih.gov/sites/entrez?db=snp&cmd=search&term=+rs111033664) | Nonsense | N/A | N/A | N/A |
| 9,34647845,1,C/T | CAC-tAC | ENST00000378842 | H132Y | EXON CDS | [rs111033688:T](http://www.ncbi.nlm.nih.gov/sites/entrez?db=snp&cmd=search&term=+rs111033688) | Nonsynonymous | DAMAGING | 0.01 | 3.01 |
| 9,34647855,1,C/T | TCG-TtG | ENST00000378842 | S135L | EXON CDS | [rs111033690:T](http://www.ncbi.nlm.nih.gov/sites/entrez?db=snp&cmd=search&term=+rs111033690) | Nonsynonymous | DAMAGING | 0.02 | 3.01 |
| 9,34647957,1,A/C | CAG-CcG | ENST00000378842 | Q169P | EXON CDS | novel | Nonsynonymous | DAMAGING | 0 | 3.01 |
| 9,34648114,1,C/A | ATC-Ata | ENST00000378842 | I170I | EXON CDS | [rs61735984:A](http://www.ncbi.nlm.nih.gov/sites/entrez?db=snp&cmd=search&term=+rs61735984) | Synonymous | TOLERATED | 1 | 3.01 |
| 9,34648116,1,T/C | TTT-TcT | ENST00000378842 | F171S | EXON CDS | [rs111033715:C](http://www.ncbi.nlm.nih.gov/sites/entrez?db=snp&cmd=search&term=+rs111033715) | Nonsynonymous | DAMAGING | 0 | 3.01 |
| 9,34648128,1,G/A | GGT-GaT | ENST00000378842 | G175D | EXON CDS | [rs111033718:A](http://www.ncbi.nlm.nih.gov/sites/entrez?db=snp&cmd=search&term=+rs111033718) | Nonsynonymous | DAMAGING | 0 | 3.01 |
| 9,34648167,1,A/G | CAG-CgG | ENST00000378842 | Q188R | EXON CDS | [rs75391579:G](http://www.ncbi.nlm.nih.gov/sites/entrez?db=snp&cmd=search&term=+rs75391579) | Nonsynonymous | DAMAGING | 0 | 3.01 |
| 9,34648376,1,C/T | CGA-tGA | ENST00000378842 | R204* | EXON CDS | [rs111033737:T](http://www.ncbi.nlm.nih.gov/sites/entrez?db=snp&cmd=search&term=+rs111033737) | Nonsense | N/A | N/A | N/A |
| 9,34648763,1,G/A | CGT-CaT | ENST00000378842 | R231H | EXON CDS | [rs111033754:A](http://www.ncbi.nlm.nih.gov/sites/entrez?db=snp&cmd=search&term=+rs111033754) | Nonsynonymous | DAMAGING | 0 | 3.01 |
| 9,34648998,T,. |  | ENST00000378842 | L275fs | EXON CDS | rs111033777 | Frameshift | N/A | N/A | N/A |
| 9,34649029,1,G/T | AAG-AAt | ENST00000378842 | K285N | EXON CDS | [rs111033773:T](http://www.ncbi.nlm.nih.gov/sites/entrez?db=snp&cmd=search&term=+rs111033773) | Nonsynonymous | DAMAGING | 0 | 3.01 |
| 9,34649050,1,G/A | ACG-Aca | ENST00000378842 | T292T | EXON CDS | [rs1055607:A](http://www.ncbi.nlm.nih.gov/sites/entrez?db=snp&cmd=search&term=+rs1055607) | Synonymous | TOLERATED | 1 | 3.01 |
| 9,34649053,1,C/T | TCC-TCt | ENST00000378842 | S293S | EXON CDS | [rs115527942:T](http://www.ncbi.nlm.nih.gov/sites/entrez?db=snp&cmd=search&term=+rs115527942) | Synonymous | TOLERATED | 1 | 3.01 |
| 9,34649442,1,A/G | AAC-gAC | ENST00000378842 | N314D | EXON CDS | [rs2070074:G](http://www.ncbi.nlm.nih.gov/sites/entrez?db=snp&cmd=search&term=+rs2070074) | Nonsynonymous | TOLERATED | 1 | 3.01 |
| 9,34649447,1,T/C | CAT-CAc | ENST00000378842 | H315H | EXON CDS | [rs61735982:C](http://www.ncbi.nlm.nih.gov/sites/entrez?db=snp&cmd=search&term=+rs61735982) | Synonymous | TOLERATED | 1 | 3 |
| 9,34649476,1,C/T | CCG-CtG | ENST00000378842 | P325L | EXON CDS | [rs111033794:T](http://www.ncbi.nlm.nih.gov/sites/entrez?db=snp&cmd=search&term=+rs111033794) | Nonsynonymous | DAMAGING | 0 | 3.01 |

Table S2-b: Results of Poliphen-2 simulations

| Substitution | Protein Acc | Prediction | Score | sensitivity | specificity |
| --- | --- | --- | --- | --- | --- |
| M1T # | P07902 | BENIGN | 0.00 | 1.00 | 0.00 |
| R33S # | P07902 | PROBABLY DAMAGING | 1.00 | 0.00 | 1.00 |
| R33H | P07902 | PROBABLY DAMAGING | 1.00 | 0.00 | 1.00 |
| P73S # | P07902 | PROBABLY DAMAGING | 0.996 | 0.55 | 0.98 |
| H132Y | P07902 | POSSIBLY DAMAGING | 0.685 | 0.86 | 0.92 |
| S135L | P07902 | POSSIBLY DAMAGING | 0.940 | 0.80 | 0.94 |
| Q169P # | P07902 | PROBABLY DAMAGING | 0.959 | 0.78 | 0.95 |
| F171S | P07902 | PROBABLY DAMAGING | 1.00 | 0.00 | 1.00 |
| G175D | P07902 | PROBABLY DAMAGING | 1.00 | 0.00 | 1.00 |
| Q188R | P07902 | PROBABLY DAMAGING | 1.00 | 0.00 | 1.00 |
| R231H | P07902 | PROBABLY DAMAGING | 1.00 | 0.00 | 1.00 |
| K285N | P07902 | PROBABLY DAMAGING | 0.996 | 0.55 | 0.98 |
| N314D | P07902 | BENIGN | 0.00 | 1.00 | 0.00 |
| P325L | P07902 | PROBABLY DAMAGING | 1.00 | 0.00 | 1.00 |

# novel missense mutations
